# Preparation and Characterization of Stable Nanoliposomal Formulation of Fluoxetine as a Potential Adjuvant Therapy for Drug-Resistant Tumors 

**Published:** 2014

**Authors:** Azadeh Haeri, Behdokht Alinaghian, Marjan Daeihamed, Simin Dadashzadeh

**Affiliations:** a *Department of Pharmaceutics, School of Pharmacy, Shahid Beheshti University of Medical Sciences, Tehran, Iran.*; b *Pharmaceutical Sciences Research Center, Shahid Beheshti University of Medical Sciences, Tehran, Iran. *

**Keywords:** Fluoxetine, Nanoliposomes, Formulation parameters, PEGylation, Stability, Drug-resistant tumors

## Abstract

Chemotherapy research highly prioritizes overcoming the multidrug resistance (MDR) in human tumors. Liposomal formulation of fluoxetine, as a fourth generation chemosensitizer, was constructed and characterized for percent entrapment, release profile, morphology, particle size, zeta potential and stability. Liposomes were prepared using different active loading techniques. The influence of different formulation variables such as loading methodology, type of main lipid, addition of PEGylated lipid and cholesterol percentage was evaluated to achieve required entrapment efficiency, in vitro release behavior and stability. The studied parameters had significant effect on physicochemical characteristics of the nanocarriers. High fluoxetine encapsulation efficiency (83% ± 3%) and appropriate particle size (101 ± 12 nm) and zeta potential (-9 ± 2 mv) were achieved for PEGylation liposomes composed of DSPE-PEG, DSPC and cholesterol at respective molar ratio of 5:70:25. An in vitro fluoxetine release of about 20% in 48 h was observed from optimum formulation. Atomic force microscopy (AFM) studies confirmed homogeneous distribution of particles and spherical shape with smooth surface. The optimum formulation was stable for 9 days when incubated at 37 °C. The results of this study are very encouraging for application of the developed fluoxetine liposomal formulation in drug-resistant tumor models.

## Introduction

Chemotherapy is the most powerful therapeutic tool against advanced and disseminated cancers. However, in the battle against cancer, development of multiple drug resistance (MDR) is one of the major hurdles against successful chemotherapy. As the term implies, MDR refers to a cross-resistance to a wide variety of structural and functional distinct drugs or chemicals, thereby rendering the tumor unresponsive to most chemotherapeutic options (1-3). The most common mechanism of MDR is the overexpression of membrane proteins such as P-glycoprotein (P-gp, ABCB1), multidrug resistance-associated proteins (MRP1, ABCC1 and MRP2, ABCC2), and breast cancer resistance protein (BCRP, ABCG2) (2, 4). P-gp, encoded by the MDR1 gene, is the most abundantly expressed drug efflux system in cell membranes, which actively extrudes a diverse range of drugs such as taxanes, anthracyclines, vinca alkaloids, podophyllotoxins and camptothecins, against a concentration gradient from the cell (5, 6). Evidence for the role of P-gp in clinical tumor resistance is supported by studies that demonstrate P-gp expression in about 40% of breast cancer samples and its correlation with decreased treatment response (7, 8). 

The well-established role of P-gp in MDR has led to a great deal of research centered on agents that reverse or modulate P-gp activity in solid tumors. Many compounds with P-gp inhibitory activity, which are sometimes referred as “chemosensitizers”, have been identified or synthesized to address this issue (9). First generation inhibitors or modulators are pharmacologically active compounds which are already approved for other indications but also able to block P-gp. These include immunosuppressants like cyclosporin A, calcium channel blockers such as verapamil and antiestrogens like tamoxifen and toremifene. However, significant immunosuppressive and nephrotoxic effects, cardiac toxicities, low potencies, poor specificity for the drug efflux transporters and low solubility at doses required for MDR reversal limit their clinical use (10, 11). Chemical derivatization of first-generation molecules and combinatorial chemistry lead to second- and third-generation chemosensitizers, such as PSC833, VX-710, and OC144–093. These advanced generation chemosensitizers are more potent and less toxic than first-generation compounds, yet some are still prone to adverse effects, poor solubility, and unfavorable changes in pharmacokinetics of the anticancer drugs (9, 11 and 12). Moreover, diversity and heterogeneity among tumors as well as patient to patient variability in responses to the same treatment indicates that more than one chemosensitizer will be needed in the clinic (12).

Fluoxetine (FLX) is a highly active and selective serotonin reuptake inhibitor used for clinical depression for more than two decades (13). Recent studies have identified its potential to reverse MDR generated by two major pump proteins P-gp and MRP1 (12, 14 and 15). It is reported that FLX can sensitize the cytotoxic potential of conventional anticancer agents in both resistant and sensitive tumor models (12). Some studies imply the potential of FLX not only as a chemosensitizer, potentiating tumor response to anticancer drugs but also as an anticancer drug. FLX alone can inhibit the growth of many cancer cell lines by inducing apoptosis such as human neuroblastoma cell lines, rat glioma cell lines and Burkitt lymphoma cells (15, 16). FLX, which has been approved for non-cancer indications and found to act as MDR modulators, can be categorized as a first-generation chemosensitizer. However, unlike the first-generation chemosensitizers, FLX exerts its ability to modulate MDR cancer cells at low doses that indicates it may merit a separate category, possibly fourth-generation chemosensitizers (12). 

In spite of high potency and specificity, a major confounding factor in use of free form of a chemosensitizer is the fact that besides exerting an effect on P-gp function in cancerous cells, it also has a profound effect on pharmacokinetics and cytotoxicity of the anticancer drugs concurrently administered (17). The pharmacokinetic interaction and increase in cytotoxicity of anticancer drugs by a MDR modulator is due to extensive biodistribution of the MDR modulators in normal organs, such as intestine, liver, kidneys, lung and brain, inhibiting P-gp function in these tissues and causing increased distribution or decreased excretion of the chemotherapeutics (18). Furthermore, nonselective distribution following administration of free drug may lead to low and sub-therapeutic drug concentration at target cancerous tissues.

A great deal of these shortcomings of chemosensitizers may be tackled by administering these agents using nanoparticulate delivery systems (19). It should be noted that encapsulation of chemosensitizers in nanocarriers in turn can lead to preferential accumulation in sites of tumor growth due to enhanced permeability and retention (EPR) effects associated with solid tumors (20). Moreover, incorporation of drug in nanoparticulate formulations can result in considerable improvements in circulation lifetimes and in vivo drug exposure and retention (20, 21). Over the past few decades, liposomes have received particular attention for the encapsulation and the controlled and prolonged delivery of active molecules to the site of action. They are attractive systems because of their biocompatible and biodegradable composition, 

their potential for entrapping both hydrophilic and lipophilic drugs, and their colloidal size which make them useful for various applications (21, 22). 

Recently, Ong and his coworkers prepared liposomal formulation of FLX (23). The prepared liposomes entraped only 70% of the added drug at lipid to drug molar ratio of 20 (23), which is a considerably high lipid concentration for remote loading methodology with limited industrial scale application. High lipid doses may raise concerns of toxicity, worsen the physical characteristics and reduce the economic feasibility of pharmaceutical scale production of the dosage form. Therefore, formulation strategies for preparation of liposomes with desirable encapsulation capacity and efficient FLX loading are still needed and should be actively explored. In light of these considerations, in the present study, formulation of stable liposomal delivery system of FLX is described. Various formulation factors, including solubility of FLX in different salt solutions, different hydration and elution buffers, main lipid, addition of PEG lipid and percentage of cholesterol which are expected to influence drug accumulation in the intraliposomal interior, release kinetics and formulation stability are taken into account.

## Experimental

*Materials*

Fluoxetine (FLX), 98% pure, was a kind gift from Dr. Abidi Pharmaceutical Co. (Tehran, Iran). Distearoylphosphatidylcholine (DSPC), dipalmitoylphosphatidylcholine (DPPC), dimyristoylphosphatidylcholine (DMPC), purified egg phosphatidylcholine (EPC) and distearoylphosphatidylethanolamine-poly(ethyleneglycol)2000 (DSPE-PEG2000) were obtained from Lipoid GmbH (Germany). Cholesterol (Chol), potassium chloride, citric acid, sodium hydroxide, disodium ethylenediaminetetraacetate (EDTA), sodium chloride, potassium dihydrogen phosphate, disodium hydrogen phosphate, ammonium sulfate, chloroform, methanol, and HPLC-grade acetonitrile were supplied by Merck (Darmstadt, Germany). 

*Solubility of FLX*

The solubility of FLX was investigated in 300 mM EDTA disodium salt, ammonium sulfate, or sodium citrate salt solutions (pH=4). The pH values of these salt solutions were adjusted with sodium hydroxide or hydrochloric acid. The salt solutions were added to FLX powder. Each sample was vortexed for 5 min and incubated at 25 °C for 12 h. The drug precipitate was separated from the supernatant by centrifugation (10,000×g, 10 min). The FLX concentration in the supernatants was quantified by high-performance liquid chromatography (HPLC) as described in section 2.4.5.

*Liposomes preparation*

FLX was remote-loaded as previously described for other weak basic drugs (24). Empty liposomes (ELs) were prepared by the lipid film hydration method. Briefly, the lipid mixture of the desired molar composition was dissolved in chloroform/methanol 4:1 (v:v). The organic solvents were removed under vacuum in a rotary evaporator (90 rpm) at 65 °C to form a thin lipid film. Evaporation was continued for 2 h after the dry residue appeared to ensure removal of all traces of solvents. The obtained thin film was then hydrated with ammonium sulfate buffer or citrate buffer (300 mM) at 65 °C for 1 h. The resulting multivesicular liposomes were extruded several times through Nuclepore polycarbonate filters (0.4, 0.2, or 0.1 μm in series, Whatman, UK) mounted in a 10-mL Lipex Thermoline extruder (Northern Lipids, Vancouver, BC, Canada) to produce samples with a narrow size distribution. The extrusion was carried out at 65 °C to maintain vesicles above phase transition temperature. The phospholipid content of the liposomes was calculated based on the method established by Stewart (25).

The resulting ELs were dialyzed for 12 h at room temperature against the external buffer to establish a pH gradient across the liposome membrane. FLX in buffer was then mixed and incubated with ELs at 60 °C for 1 h. Residual nonencapsulated FLX were removed by exhaustive dialysis against phosphate-buffered saline (PBS) for 12 h at 4 °C. *Liposome characterization*

The prepared liposomes were characterized with respect to encapsulation efficiency (EE), size distribution, zeta potential, stability, morphology and in vitro release kinetics.

*Estimation of encapsulation efficiency*

EE of liposomes was determined using the dialysis technique for separating the non-entrapped drug from liposomes. Small aliquot of liposomes (50 μL) was diluted in 950 μL methanol, and then it was subjected to sonication until complete liposomes disruption. Quantitative determination of FLX was performed by a validated HPLC method developed in our laboratory (See section 2.4.5). Lipid components did not have any interference with the estimation of FLX. The EE was calculated from the ratio of the FLX content of the liposomes and the amount of FLX used for preparation and expressed as a percentage.

*Determination of particle size and zeta potential*

The mean hydrodynamic diameter and polydispersity of liposomes were determined by the dynamic light scattering technique (DLS) in a Zetasizer Nano ZS (Malvern Instruments, CO., UK) equipped with a 633 nm laser source. The analysis was performed at 25 °C and a scattering angle of 90° after the appropriate dilution with PBS. Each value given is the average of three measurements.

Zeta potential of the liposomes was measured using electrophoretic light scattering by a Malvern Zetasizer Nano ZS. The measurement was performed at 25 °C after appropriate dilution with distilled water. All of the measurements were repeated at least three times.

*Stability of formulations*

The stability of liposomal formulations was evaluated at 4 °C and 37 °C. At intervals aliquots of samples were withdrawn for EE, particle size and zeta potential measurement.

*In-vitro drug release*

The in vitro drug release of FLX encapsulated in different liposomal formulations was determined using dialysis tubing (molecular weight cutoff of 12 KDa). Liposomal solutions (0.5 mL) were placed in dialysis bag and immersed in 20 mL of release medium (PBS), under 100 rpm magnetic stirring at 37 °C. At predetermined time intervals, 1 mL samples were withdrawn and immediately replaced with an equal volume of the fresh medium. The concentration of FLX in the samples was measured by HPLC as described below (See section 2.4.5). The cumulative percentage of drug release was calculated and plotted versus time. 

*Analysis of FLX by HPLC*

The amount of FLX in nanoformulations and in release medium was determined by reverse-phase HPLC. The chromatographic separation was accomplished on a PerfectSil C18 column, (4.6 × 150 mm, 5 μm particle size, MZ-Analysentechnik, Mainz, Germany) using an isocratic mobile phase that consisted of acetonitrile and water (50:50, v/v, pH=3.5) as the eluent at a flow rate of 1 ml/min (K-1001 solvent delivery pump, Knauer, Germany). The analyte was detected with the ultraviolet detection (K-2600 UV detector, Knauer, Germany) at the wavelength of 227 nm. The column was kept at room temperature. A linear response was observed in the range of 100–1000 ng/mL, with a correlation coefficient, of 0.9995. The coefficients of variation for the inter-day and intra-day assays were found to be less than 5.0%. 

*Imaging of liposomes by atomic force microscopy*

The shape and morphology of empty and drug-loaded liposomal formulations were observed using a NanoWizard®II atomic force microscope (AFM, JPK Instruments, Germany). Before AFM imaging, nanoformulations were diluted with distilled water and one drop of diluted dispersion was mounted on the glass slides, air-dried and scanned by the AFM. AFM was operated at room temperature with image resolution of 512 pixels × 512 pixels at a scan speed from 0.9 to 1.2 Hz in air. 

*Statistical analysis*

Statistical analysis was performed using SPSS software (version 11.5, SPSS, Chicago, IL). Data are expressed as mean ± SEM. Student›s t test was used to compare each variable between two groups. The analysis of variance (ANOVA), which was followed by the post-hoc test, was used when more than two groups were compared. For all of the tests, the differences of P < 0.05 were interpreted as statistically significant. 

## Results and Discussion

Active loading techniques provide a versatile method to prepare liposomes with encapsulated cargo, provided that factors influencing drug loading, release properties and formulation stability are systematically studied. To the best of our knowledge, no efforts have been made to evaluate effect of formulation parameters such as lipid composition, presence of PEG and Chol/main lipid ratio on physicochemical characteristics of FLX nanoliposomes. In our study, EE, as a key parameter in liposomal drug delivery, was chosen as one of the major parameters to be optimized. 

Besides efficient loading, release of the drug which is a critical factor for a drug delivery system, was also studied.

Based on our preliminarily studies, encapsulation of FLX in preformed liposomes led to significant increase in vesicle size (especially following incubation at 37 °C) and within a few hours aggregate of drug molecules and lipidic components appeared suggesting the occurrence of a phase separation phenomenon which was in agreement with the previous report of interaction of FLX with pure as well as cholesterol containing phosphatidylcholine membranes (26). Therefore, in present study we closely monitored liposome size at 37 °C. 

*Solubility of FLX in different salt solutions*

One of the main goals in active loading techniques is to achieve drug precipitation in the form of a low solubility salt inside the liposomes, and thereby obtain controlled *in-vivo *drug release from liposomal nanocarriers (24). In the case of FLX, formation of poorly soluble drug complexes inside liposomes may contribute to lower level of drug bilayer interactions and increased liposomal drug retention properties. This speculation was strengthened by observations of other relatively hydrophobic drugs such as idarubicin, vinorelbine and ciprofloxacin, for which drug bilayer interactions led to rapid drug leakage (27-29).

Previous experiments showed that low solubility precipitates formed from sulfate, citrate and EDTA with weakly basic drugs like anthracyclines and this strategy was used for active drug loading (24, 27). In this study solubility of FLX in different salt solutions was examined. As shown in Table 1, FLX exhibits low solubility at 25 °C in 300 mM ammonium sulfate, citrate and sodium EDTA solutions (pH≈4). The solubility of FLX in sulfate and citrate solutions at pH 4.0 was lower than sodium EDTA solution which is in agreement with reports on the solubility of lipophilic cationic drugs in these solutions (24). The solubility of FLX in EDTA solution is relatively low (compered to normal saline and PBS), but it is about two to three times higher than the FLX solubility in sulfate and citrate solutions (Table 1).

**Table 1:** Solubility of FLX in different salt solutions (n=3, mean ± SEM).

**Buffer (300 mM)**	**Solubility ** **(mg /mL of buffer)**
Citrate buffer	1.2 ± 0.2
Ammonium sulfate	1.9 ± 0.3
Sodium EDTA	4.1 ± 0.3

Loading of weakly basic drugs by means of salt gradients includes two synergistic effects, both of which result in the fact that basic drugs can no longer pass the lipid membrane. On the one hand loading is driven by protonation and charging of the drugs within the liposome interior phase, and on the other by precipitation of cargo within aqueous core of the vesicle (24, 30). In contrast, in case of high drug solubility in internal liposome phase, the amount of free drug that is not precipitated inside the liposomes should be relatively high, facilitating drug partitioning into the lipid bilayer and consequent drug-lipid interaction.

**Figure 1:**
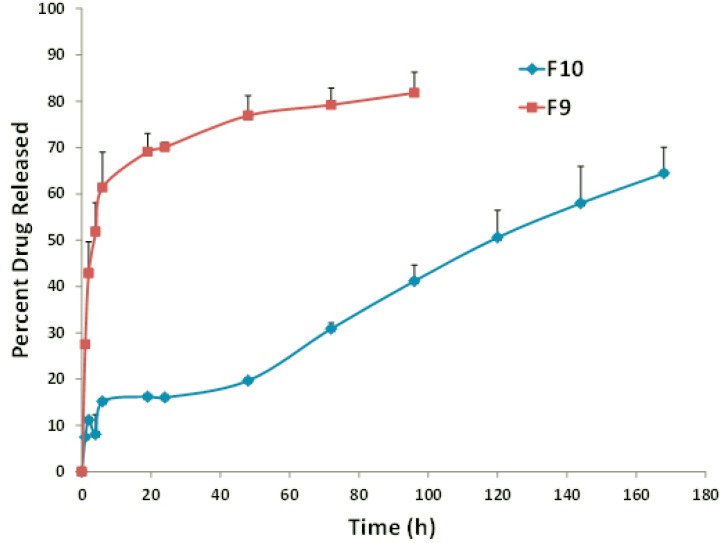
*In-vitro *release profile of FLX from F9 (DPPC:Chol:PEG 70:25:5) and F10 (DSPC:Chol:PEG 70:25:5) liposomal formulations. Data represent mean ± SEM (n=3).

*Effect of hydration and elution buffers on physicochemical characteristics of FLX liposomes*

To assess and compare the EE, release behavior and stability of the FLX-bearing liposomes loaded via transmembrane gradients, two different liposomal formulations were prepared using sodium citrate buffer and ammonium sulfate gradient based on the lower solubility results of these solutions (Table 2, F1 and F2 Formulas). Comparing to citrate loading method, ammonium sulfate technique appears to marginally improve loading efficiency with comparable stability and release profile. The fact that sulfate is a salt of a stronger acid as compared to citrate, might explain the minor difference in the EE of these two loading procedures. The ammonium sulfate loading procedure was first described for liposomal encapsulation of doxorubicin by the Barenholz group (24, 31). Following encapsulation of (NH4)2SO4, the external solution is exchanged for an iso-osmotic solution to establish a stable (NH4)2SO4 gradient. Due to the high permeation coefficient of ammonia (1.3×104 cm/s) as compared to the permeation coefficient of protons (10−3 to 10−8 cm/s), NH3 readily crosses the liposome bilayer, leaving behind one proton for every molecule of ammonia lost. This phenomenon results in acidification of the liposome interior phase which contributes to improved loading capacity of weak basic molecules (24, 31).

**Table 2:** Effect of hydration and elution buffers on entrapment efficiency and release profile of FLX loaded EPC/Chol (75:25) liposomes (n = 3, mean ± SEM).

**Formulation**	**Hydration buffer**	**Elution buffer**	**EE%**	**Stability at 37 °C**	**%DR**1h	**%DR**6h	**%DR**24h	**%DR**48h
F1	Citrate buffer	PBS (pH=7.4)	66.1 ± 1.1	48 h	10.6 ± 0.1	17.5 ± 1.8	35.2 ± 1.0	50.2 ± 2.1
F2	Ammonium sulfate	PBS (pH=7.4)	70.2 ± 1.2	48 h	7.7 ± 0.6	23.2 ± 2.0	38.5 ± 1.5	52.8 ± 1.9
F3	Ammonium sulfate	PBS (pH=8.5)	72.4 ± 1.3	48 h	8.1 ± 0.9	25.2 ± 3.1	40.5 ± 2.6	53.5 ± 1.5

It was reported that the initial ΔpH values between internal and external buffers could influence drug accumulation in liposomes (27). The effect of the external PBS pH on FLX encapsulation was then evaluated while the internal pH was kept constant. The results showed that increasing external pH to 8.5 did not have any significant effect on physicochemical characteristics of FLX liposomes (Table 2, F2 and F3 Formulas). Due to incompatibility issues, higher external pHs were not studied.

*Effect of lipid composition on physicochemical characteristics of FLX liposomes*

Encapsulation of a drug depends, to a large degree, on the lipid composition (32-35). Further, experiments were set up to investigate the effect of the different lipidic compositions upon the FLX loaded vesicles. The selection of lipids for preparing liposomes as a drug delivery system depends on many factors, including entrapment efficiency, availability, cost, safety, and ease of utilization of the lipids. In this study, in order to investigate the role of phospholipid composition on physicochemical characteristics of liposomes, different phospholipids (Table 3), which vary in acyl chain length, the degree of saturation of the acyl chains and bilayer fluidity, were used for preparing different liposomal formulations. The chosen lipids are the most common used lipids for the liposome preparation. As (NH4)2SO4 buffer enabled the highest drug accumulation (Table 2), this buffer was selected for further experiments. All formulations presented in Table 4 prepared at fixed lipid/drug ratio (10) and Chol:lipid molar ratio (25:75). From the results, it can be readily noticed that EE, drug release rate and stability of the nanocarrier closely correlated to the liposomal lipid composition.

**Table 3:** Liposomal phospholipids used in this study and their gel-to-liquid crystalline phase transition temperatures.

**Phospholipids**	**Acyl chain length, No. of unsaturation**	**Transition temperature (Tm °C)**
EPC	Mixture†	-2.5
DMPC	14:0	23
DPPC	16:0	41
DSPC	18:0	55

**Figure 2:**
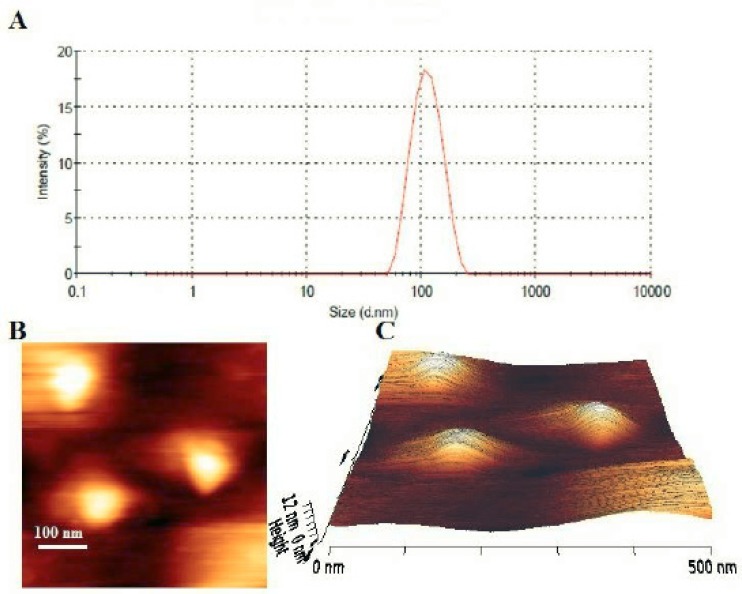
A) Particle size distribution profile of fluoxetine loaded PEGylated DSPC liposomes. B) Two-dimensional AFM image of fluoxetine loaded PEGylated DSPC liposomes. Bar, 100 nm. C) Three-dimensional AFM image of fluoxetine loaded PEGylated DSPC liposomes

Overall, a higher extent of incorporation and stability was observed for the DSPC liposomes when compared to the EPC-, DMPC- and DPPC-vesicles. Increase in the fatty acid chain length of DSPC and the gel state of liposomes composed of this lipid are probable responsible factors (21, 36). In the presence of rigid acyl chain of DSPC, freedom of movement of lipophilic chains decreases and this may lead to lower drug-membrane interaction and higher stability of the formulation. In vitro release assays appears to be dependent on the main lipid and, interestingly, DSPC formulations exhibited improved drug retention and sustained drug release of about 34% during 48 hours (Figure 2, formulation F10) which was well correlated with stability results at 37 °C. It might be explained by the high rigidity of bilayer membrane, which minimizes the leakage of entrapped materials (21, 36 and 37).

It is worth noting that DPPC:Chol FLX liposomes showed the lowest EE and the highest drug release rate (Figure 2, formulation F9) when compared to liposomes prepared from the other main lipids (Table 4). The main lipid composition of F5 was DPPC with C16 fully-saturated acyl chain and phase transition temperature of approximately 41.5–41.9 °C (Table 3, 36). When the temperature elevated to 37 °C, membrane permeability rate increased, displaying the burst increase in percentage of FLX released. This is in agreement with other published findings which have also shown temperature sensitive liposomes, prepared from DPPC as the only main lipid, was highly unstable under physiological conditions (38).

**Table 4:** Effect of lipid composition on entrapment efficiency and release profile of FLX loaded liposomes (n=3, mean ± SEM).

**Formulation**	**Lipid composition**	**EE%**	**Stability at 37 °C**	**%DR**1h	**%DR**6h	**%DR**24h	**%DR**48h
F2	EPC:Chol 75:25	70.2 ± 1.2	48 h	7.7 ± 0.6	23.2 ± 2.0	38.5 ± 1.5	52.8 ± 1.9
F4	DMPC:Chol 75:25	56.6 ± 0.2	72 h	39.1 ± 2.5	56.1 ± 0.7	56.0 ± 2.2	58.7 ± 2.4
F5	DPPC:Chol 75:25	50.5 ± 1.9	72 h	60.1 ± 1.9	70.3 ± 3.6	69.7± 0.1	72.1 ± 5.0
F6	DSPC:Chol 75:25	72.5 ± 1.1	96 h	22.2 ± 1.5	28.9 ± 2.5	30.4 ± 2.4	34.0 ± 4.7

*Effect of PEG-lipid on physicochemical characteristics of FLX liposomes*

Polyethylene glycol (PEG) modification on the liposomal surface is known to be effective in increasing formulation stability, controlling release rate, prolonging blood circulation time and preventing carrier uptake by the reticuloendothelial system. These properties made PEGylated liposomes an attractive platform to improve the therapeutic index of a variety of drugs. The previous studies have suggested that 1–5 mol% DSPE-PEG2000 is commonly added to liposomal formulations and 5 mol% DSPE-PEG2000 has been reported as the sufficient and optimal concentration (21, 36 and 39). After incorporating 5 mol% of PEGylated component, most of liposomes displayed higher entrapment capacity (1-10% based on the type of main lipid) than conventional non-PEGylated ones (Table 5). This observation was in agreement with studies reported the enhancing role of PEG in obtaining high loading efficiency (40, 41). Here, the EE for F10 formula was significantly higher than that reported by the previously published study on FLX liposomal formulation (maximum EE was approximately 70%) (23). In the mentioned study, the parameter of L/D molar ratio was the only formulation factor that was evaluated to determine the optimum formulation and the maximum EE was achieved at L/D molar ratio of 20 which was two- fold higher than that used in the present study.

**Table 5:** Effect of lipid composition and PEG-lipid on entrapment efficiency and release profile of FLX loaded liposomes

**Formulation**	**Lipid composition**	**EE%**	**Stability at 37 °C**	**%DR**1h	**%DR**6h	**%DR**24h	**%DR**48h
F7	EPC:Chol:PEG 70:25:5	77.3 ± 1.4	72 h	13.5 ± 1.3	31.1 ± 1.4	34.6 ± 1.5	52.5 ± 3.2
F8	DMPC:Chol:PEG 70:25:5	63.3 ± 1.6	96 h	42.1 ± 2.8	53.1 ± 1.1	55.2 ± 1.2	69.3 ± 4.4
F9	DPPC:Chol:PEG 70:25:5	51.7 ± 1.2	96 h	27.5 ± 0.1	61.4 ± 7.6	70.1 ± 1.1	76.9 ± 4.3
F10	DSPC:Chol:PEG 70:25:5	83.0 ± 3.4	216 h	8.1 ± 0.1	15.9 ± 0.2	16.6 ± 0.1	20.2 ± 0.5

The drug release rate of conventional and PEGylated EPC- and DMPC-liposomes were almost comparable. In case of DPPC and DSPC liposomes, the release rate of the drug was higher in conventional liposomes than that of stealth carriers. It seems that characteristics of main lipid can markedly influence the capacity of PEG coated liposomes in modulating drug release rate. 

PEGylated liposomes containing FLX were evaluated for physical stability at 37 °C. The physical stability was evaluated by monitorin vesicle size as previously described. PEGylated DSPC liposomes (F10) were stable for about 9 days at 37 °C, and retained about 80% of their initial drug content over 48 hours. The PEG chains on the nanocarrier surface have a higher volume of hydrated layer owning to their hydrophilicity. This can contribute to improved colloidal stability through both steric repulsion by PEGylated lipids on the surface of the particles as well as electrostatic repulsion by the ions in the hydrated layer thus retarding the increase in the nanoparticle size (42).

*Effect of cholesterol percentage on physicochemical characteristics of FLX liposomes*

Chol plays a critical role in liposome composition. In the literature it has been extensively reported that the formation and stability of the liposomes are highly dependent on the phospholipid-to-Chol ratio and it has great impact on the in vitro and *in-vivo *behavior of the carrier. Chol is a common component of liposomes, controlling membrane permeability, providing rigidity to the membrane, stabilizing the bilayer structure and improving plasma stability (21, 36 and 43). Despite these well-recognized effects of Chol in conventional liposomes, recent research also focuses on low Chol or Chol free liposomes especially for incorporation of hydrophobic molecules such as sirolimus (33), estradiol (34) and idarubicin (44). Therefore, besides phospholipids modification, the effect of Chol was investigated by varying the DSPC-to-Chol ratio keeping the total lipid constant (Table 6).

**Table 6:** Effect of cholesterol percentage on entrapment efficiency and release profile of FLX loaded liposomes (n=3, mean ± EM).

**Formulation**	**Lipid composition**	**EE%**	**Stability at 37 °C**	**%DR**1h	**%DR**6h	**%DR**24h	**%DR**48h
F11	DSPC:Chol 60:40	80.1 ± 1.8	96 h	28.6 ± 1.3	34.6 ± 2.3	35.1 ± 1.4	44.1 ± 1.6
F6	DSPC:Chol 75:25	72.5 ± 1.1	96 h	22.2 ± 1.5	28.9 ± 2.5	30.4 ± 2.4	34.0 ± 4.7
F12	DSPC:Chol 90:10	69.5 ± 2.1	96 h	10.3 ± 0.5	19.8 ± 0.9	21.2 ± 1.5	28.4 ± 2.1

No significant difference was noticed in EE of the formulation by increasing Chol percentage from 10% to 25%. Further increase in Chol level to 45% resulted in significant improvement in drug loading (Table 6). During loading procedure (above transition temperature of the main lipid), Chol could alter the fluidity of the phospholipid chains by increasing the microviscosity of liposomal membrane conferring more rigidity (45, 46), preventing leakage of the encapsulated drug out of vesicle which consequently led to the greater drug entrapment. 

In order to ascertain the effect of Chol percentage on the drug release and stability of liposomes, in vitro release study was conducted for F6, F11 and F12 (Table 6). We could observe a direct relation between the level of Chol and drug release, the higher Chol percentage the faster the drug release. Compared with F11, the release profiles of F6 and F12 formulations were obviously delayed. The cumulative drug released from 10%, 25% and 45% Chol containing formulations were found to reach 28%, 34% and 44% during 48 hours, respectively (Table 6). In release studies, which was performed well below transition temperature of DSPC liposomes, Chol probably increase the fluidity of the long acyl chain phospholipid bilayer (45, 46), resulted in leakage and permeability of drug at 37 °C.

It was concluded that liposomal formulation with 25% Chol content was more beneficial for the efficient encapsulation as well as to achieve a controlled release behavior. Extra Chol was unfavorable due to stimulating burst drug release.

*Particle size, zeta potential and morphology*

The hydrodynamic diameters of the liposomes were measured by the dynamic light scattering method. All of the formulations had a particle size distribution from 90 to 125 nm (Figure 2A), which were consistent with results expected for vesicles extruded through filters with 100 nm pore size. The polydispersity index (<0.2) indicated that different formulations formed narrowly-dispersed nanostructures without any aggregation in water. However, in F1, F2 and F3 formulations after 48 hours incubation at 37 °C, significant increase in vesicle mean size and PDI were demonstrated (Table 2). The liposomal preparations generally showed a rather constant particle size and PDI during the loading process. 

The zeta potential is the electrostatic charge of the particle surface which acts as a repulsive energy barrier controlling the stability of dispersion and opposing the proximity of particles and aggregation. The zeta potential of liposomes in the absence of DSPE-PEG was in the range of −1 to −3 mv. Addition of PEG-lipid increased the negativity of the surface charge of the carrier. The zeta potential of the drug-loaded PEGylated nanoliposomes ranged from −8 to −13 mv. 

The prepared stealth liposomal formulation (F10) was studied under AFM for morphological evaluation. The results showed uniform, homogenous and spherical shape vesicles with smooth surface (Figures 2B and 2C). No aggregation or fusion of the vesicles was observed. The observed liposomes had sizes around 100 nm which was in rather good agreement with the results of dynamic light scattering measurements.

*Liposome stability*

The physical and chemical stability of F10 Formula were evaluated at 5 °C, 25 °C and 37 °C for two weeks. The stealth DSPC liposomes loaded with FLX were stable for at least two weeks at room and refrigerator temperatures as the particle size and the EE of the liposomes did not change significantly during this period (data not shown). The data presented in Table 5 demonstrate that when F10 formula was stored at 37 °C, the particle size of the carrier did not change significantly during 9 days period.

## Conclusion

This study contributes to the understanding of the different formulation parameters on FLX loading into liposomes. The studies presented here suggested that the counter ion, the main lipid, the Chol percentage and addition of PEG lipid play important roles in drug loading efficiency, stability and release kinetics of FLX loaded liposomes. Formulation of highly stable nanoliposome constructs of FLX was prepared and characterized regarding various *in-vitro *characteristics. Nanosized PEGylated DSPC liposomes would be promising delivery systems for FLX in the treatment of drug-resistant tumors. Further *in-vivo *studies in tumor models need to be warranted in order to derive the feasibility of these formulations to improve the therapeutic efficacy.
